# Predictive value of hematological parameters in testicular salvage: A 12-year retrospective review

**DOI:** 10.3389/fped.2022.989112

**Published:** 2022-08-17

**Authors:** Pengyu Chen, Weipeng Huang, Lei Liu, Nana Chen, Guanglun Zhou, Mengkui Sun, Shoulin Li

**Affiliations:** ^1^Department of Urology, Shenzhen Children's Hospital, China Medical University, Shenzhen, China; ^2^Department of Critical Care Medicine, Zhongnan Hospital, Wuhan University, Wuhan, China; ^3^Department of Urology, Shenzhen Children's Hospital, Shenzhen, China

**Keywords:** ischemia-reperfusion, monocyte, orchiectomy, testicular salvage, testicular torsion

## Abstract

**Purpose:**

This study aimed to evaluate the predictive value of preoperative hematological parameters for testicular salvage in patients with testicular torsion.

**Methods:**

Clinical data of patients with testicular torsion treated at Shenzhen Children's Hospital from January 2010 to December 2021 were analyzed retrospectively. The data collected included age, symptom duration, degree of spermatic cord torsion, the surgical approach adopted, hematological parameters, and ultrasound results during postoperative follow-up.

**Results:**

The study participants were classified into three groups as follows: the successful testicular salvage group (*n* = 43), failed testicular salvage group (*n* = 124), and control group (*n* = 100). Univariate analysis showed that testicular salvage was related to patient age, duration of symptoms, spermatic cord torsion degree, white blood cell count, lymphocyte count, monocyte count, platelet-lymphocyte ratio, and neutrophil-lymphocyte ratio. However, multivariate analysis revealed that symptom duration (OR = 0.948, *P* < 0.001), degree of spermatic cord torsion (OR = 0.994, *P* < 0.001), and monocyte count (OR = 0.020, *P* = 0.011) were independent risk factors for testicular torsion salvage. The monocyte count in the failed salvage group was significantly higher than in the successful salvage and control groups (*P* < 0.01).

**Conclusion:**

Monocyte count is an independent predictor of testicular salvage. Therefore, clinicians can predict the success rate of testicular salvage in patients with testicular torsion based on the monocyte count.

## Introduction

Testicular torsion is a pediatric urological emergency manifested by the rotation of the testis and epididymis around the spermatic vessels, resulting in an acute interruption of blood supply to the testis. Torsion can cause ischemic damage to the testis or even testicular necrosis if not promptly corrected. The incidence of testicular torsion in patients under 18 years of age is ~3.8/100,000. It is distributed across all age groups, with peaks in 0–12 months and 13–16 years (1, 2). The “golden time” to save the testicle after testicular torsion is 4–8 h (3). In patients with suspected testicular torsion, early surgical exploration should be performed to assess the viability of the torsioned testis before further consideration of orchiopexy or orchiectomy. Studies have reported that hematologic parameters can be used in the differential diagnosis of testicular torsion and even to predict testicular viability in the presence of testicular torsion using specific parameters (4–9). This study collected clinical data spanning over 10 years from patients with testicular torsion at our hospital, focusing on the predictive value of hematological parameters for testicular viability after testicular torsion in children.

## Materials and methods

### Patient selection and treatment measures

The medical records of children with testicular torsion who were admitted to our hospital between January 2010 and December 2021 were retrospectively collected for analysis. The inclusion criteria were: physical examination by a urologist and ultrasonography performed by an ultrasonographer in our hospital before surgery, testicular torsion confirmed by surgery, and postoperative follow-up for more than 6 months. In contrast, the exclusion criteria were parents' refusal to undergo surgical treatment, testicular torsion in newborns, and children with organ dysfunction or malignant tumors. One hundred healthy children with normal physical examination results in our hospital were randomly selected as the control group. All children in our clinical research with acute scrotal pain underwent a careful external genital physical examination, ultrasound, and routine blood examination immediately after the emergency outpatient visit. In cases where testicular torsion was suspected on ultrasound, an immediate surgical investigation was performed by an experienced urologist. The testicle was lifted from the sheath and quickly repositioned during the procedure. Furthermore, the testes were warmed in water for 20 min, if the color of the testicles turned ruddy, orchiopexy was considered. If the testes were still purple or black, the tunica albuginea was incised, and testicular viability was determined by measuring the blood seepage from the incision. Orchiopexy or orchiectomy was performed in the presence or absence of fresh blood flow within 10 min of incision.

### Variables obtained

The children's medical records were carefully reviewed to obtain information on age, duration of symptoms (time between the onset of symptoms and the start of surgery), degree of spermatic cord torsion, the surgical approach adopted, hematological parameters, and ultrasonography. Hematological parameters included white blood cell (WBC) count, neutrophil count, lymphocyte count, monocyte count, platelet count, and mean platelet volume (MPV). The neutrophil-lymphocyte ratio (NLR) and platelet-lymphocyte ratio (PLR) were calculated using blood test results.

### Grouping design

According to previous reports, the incidence of atrophy after orchiopexy in patients with testicular torsion ranges from 9.1 to 56.6% (10, 11). Therefore, the authors concluded that assessment of testicular viability should be combined with its long-term prognosis, defining orchiectomy patients and those with testicular atrophy after orchiopexy as having failed testicular salvage.

This study monitored the prognosis of children with testicular torsion through postoperative follow-up. Referring to the previous literature, patients with orchiopexy with a testicular volume difference of ≥50% between the affected testis and the contralateral testis were defined as having testicular atrophy (12–14). Patients with testicular atrophy were included in the failed testicular salvage group with those who underwent orchiectomy.

Patients with a testicular volume difference of <50% were included in the successful testicular salvage group, and healthy patients were included in the control group.

### Statistical analysis

Data following normal distribution weres assessed using the Kolmogorov–Smirnov test. For data with non-normal distribution, the non-parametric Kruskal-Wallis test was used for comparisons between groups. For a normal distribution, univariate analysis of variance (ANOVA) was used for comparison between groups. The data with non-normal distribution are expressed as medians with corresponding 25th and 75th percentiles. However, data with normal distribution are expressed as means with standard deviations. The significance level was set at *p* < 0.05. Receiver operating characteristic (ROC) curve analysis was used to assess the area under the curve (AUC) and identify optimal cut-off values. Data were analyzed using IBM SPSS 22.0 (Chicago, IL, USA).

### Ethics statement

The Institutional Review Board (IRB) approved the study protocol of the Ethics Board of Shenzhen Children's Hospital (IRB No. 2022014) and confirmed the provision of informed consent.

## Results

In total, 267 children were enrolled in this study. The first group included 167 children with testicular torsion, with a median age of 118.93 months (44.97–153.30), and the second group was a control group with a median age of 89.70 months (77.53–114.84). Table 1 compares hematological parameters between children with testicular torsion and healthy children. Significant differences in WBC, neutrophils, lymphocytes, monocytes, eosinophils, NLR, and PLR between testicular torsion children and healthy children were observed (*P* < 0.05).

**Table 1:** Baseline characteristics of patients with testicular torsion and healthy children.

	**Testicular torsion** **(*n* = 167)**	**Control** **(*n* = 100)**	***P***
Age (m)	118.93 (44.97–153.30)	89.70 (77.53–114.84)	0.062
WBC (10^9^/L)	11.36 (9.44–14.05)	7.04 ± 1.43	<0.001
Neutrophil (10^9^/L)	7.06 (4.98–9.90)	3.13 ± 0.96	<0.001
Lymphocyte (10^9^/L)	2.64 (1.71–4.40)	3.14 ± 0.84	0.043
Monocyte (10^9^/L)	0.61 (0.44–0.82)	0.37 (0.32–0.44)	<0.001
Eosinophil (10^9^/L)	0.10 (0.03–0.25)	0.28 (0.13–0.45)	<0.001
Basophil (10^9^/L)	0.04 (0.02–0.05)	0.04 (0.02–0.05)	0.932
MPV (fl)	9.75 ± 0.92	9.84 ± 0.96	0.717
Platelet (10^9^/L)	318.00 (270.00–380.00)	320.32 ± 67.17	0.379
PLR	122.22 (77.49–170.26)	107.68 ± 32.36	0.038
NLR	2.39 (1.28–5.11)	0.92 (0.76–1.32)	<0.001

WBC, white blood cell; MPV, mean platelet volume; PLR, platelet-lymphocyte ratio; NLR, neutrophil-lymphocyte ratio.

In this study, children with testicular torsion were classified into two groups: the successful testicular salvage group, comprising 43 children with good testicular development after orchiopexy, and the failed testicular salvage group, including 117 children who underwent orchiectomy and seven children with testicular atrophy after orchiopexy. There were significant differences in age, duration of symptoms, and degree of testicular torsion between the successful and failed testicular salvage groups (*P* < 0.05). Additionally, the two groups showed significant differences in WBC, lymphocyte, monocyte, PLR, and NLR (Table 2).

**Table 2:** Baseline characteristics of testicular torsion patients.

	**Total** **(*n* = 167)**	**Failed salvage** **(*n* = 124)**	**Successful salvage** **(*n* = 43)**	***P***
Age (m)	118.93 (44.97–153.30)	88.95 (22.17–152.96)	137.47 (91.00–154.73)	0.019
Symptom duration (h)	31.00 (12.00–69.00)	53.00 (26.00–75.00)	5.00 (3.00–12.00)	<0.001
Torsion degree (°)	540.00 (360.00–720.00)	540.00 (360.00–720.00)	360.00 (180.00–540.00)	<0.001
WBC (10^9^/L)	11.36 (9.44–14.05)	11.72 (9.88–14.05)	10.78 ± 4.18	0.017
Neutrophil (10^9^/L)	7.06 (4.98–9.90)	7.46 ± 3.13	6.62 (4.45–9.82)	0.546
Lymphocyte (10^9^/L)	2.64 (1.71–4.40)	2.75 (1.94–4.97)	2.08 (1.41–3.26)	0.002
Monocyte (10^9^/L)	0.61 (0.44–0.82)	0.68 (0.53–0.93)	0.39 (0.29–0.64)	<0.001
Eosinophil (10^9^/L)	0.10 (0.03–0.25)	0.10 (0.04–0.30)	0.10 (0.02–0.21)	0.212
Basophil (10^9^/L)	0.04 (0.02–0.05)	0.04 (0.02–0.06)	0.03 (0.02–0.05)	0.460
MPV (fl)	9.75 ± 0.92	9.76 ± 0.87	9.72 ± 1.06	0.699
Platelet (10^9^/L)	318.00 (270.00–380.00)	321.50 (275.50–393.25)	309.00 (264.00–357.00)	0.204
PLR	122.22 (77.49–170.26)	116.95 (69.95–150.40)	144.62 (102.81–100.47)	0002
NLR	2.39 (1.28–5.11)	2.27 (1.16–4.76)	3.32 (1.65–7.68)	0.042

WBC, white blood cell; MPV, mean platelet volume; PLR, platelet-lymphocyte ratio; NLR, neutrophil-lymphocyte ratio.

**Figure 1:**
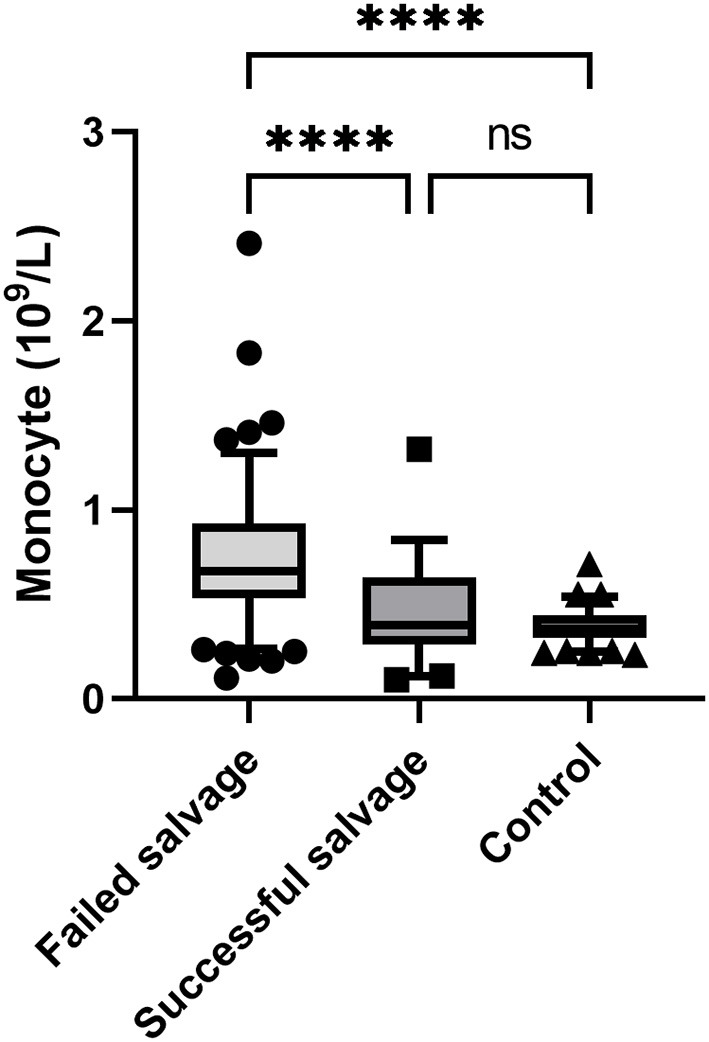
Comparison of monocyte count among the three study groups. ****, *P* < 0.001; ns, non significant.

Univariate analysis showed that testicular salvage was related to patient age, duration of symptoms, torsion degree, WBC count, lymphocyte count, monocyte count, PLR, and NLR. However, the multivariate analysis revealed that symptom duration (odds ratio [OR] = 0.948, *P* < 0.001), torsion degree (OR = 0.994, *P* < 0.001), and monocyte count (OR = 0.020, *P* = 0.011) were independent risk factors for testicular torsion salvage (Table 3). The monocyte count in the failed salvage group was significantly higher than that in the successful salvage and control groups (*P* < 0.01) (Figure 1).

**Table 3:** Univariate and multivariate analyses results.

**Variable**	**Univariate analysis**			**Multivariate analysis**		
	**OR**	**95%CI**	***P***	**OR**	**95%CI**	***P***
Age (m)	1.009	1.003–1.016	0.006	1.013	0.999–1.027	0.077
Symptom duration (h)	0.925	0.897–0.954	<0.001	0.948	0.920–0.976	<0.001
Torsion degree (°)	0.995	0.993–0.997	<0.001	0.994	0.991–0.997	<0.001
WBC (10^9^/L)	0.889	0.799–0.989	0.031	1.069	0.801–1.427	0.650
Lymphocyte (10^9^/L)	0.785	0.648–0.951	0.013	1.444	0.868–2.402	0.157
Monocyte (10^9^/L)	0.013	0.002–0.080	<0.001	0.020	0.001–0.404	0.011
PLR (10^9^/L)	1.007	1.003–1.011	0.001	1.009	0.999–1.020	0.075
NLR (10^9^/L)	1.130	1.021–1.249	0.018	0.879	0.600–1.288	0.509

OR, odds ratio; CI, confidence interval; WBC, white blood cell; PLR, platelet-lymphocyte ratio; NLR, neutrophil-lymphocyte ratio.

**Figure 2:**
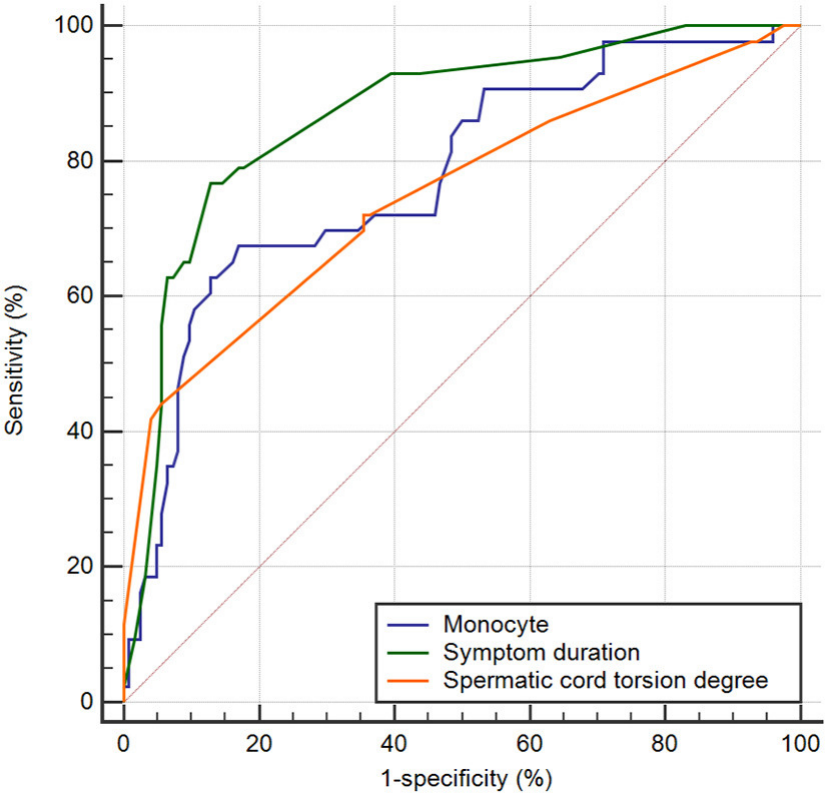
ROC curves of the monocyte, symptom duration, and torsion degree for predicting testicular salvage.

The ability of hematological parameters to predict testicular viability after testicular torsion was further evaluated using a ROC curve. ROC analysis revealed that the AUC of monocytes was 0.777 (*P* < 0.001, sensitivity 83.1%, and specificity 64.7% at a cut-off of 0.48 × 109/L), the AUC of spermatic cord torsion degree was 0.750 (*P* < 0.001, sensitivity 94.4% and specificity 44.2% at a cut-off of 270°), and that of symptom duration was 0.869 (*P* < 0.001, sensitivity 87.1% and specificity 76.7% at a cut-off of 12 h) (Figure 2). A pairwise comparison of the ROC curve showed that symptom duration and torsion degree were statistically significant (*P* = 0.032).

## Discussion

Testicular torsion is a urological condition. If not diagnosed and promptly treated, it can lead to testicular ischemia and even testicular necrosis. Many studies have shown that the survival rate after testicular torsion is related to the symptom duration and degree of testicular torsion (15). After univariate and multivariate logistic regression analyses, Zheng et al. documented that the echoes and blood flow signals of testicular parenchyma examined by ultrasound could be used as predictors of testicular torsion (16). However, an ultrasonic examination has no strict quantitative index, and it is greatly influenced by the operator's technical level and does not have a strong predictive efficiency.

In recent years, some studies have indicated that hematological parameters can predict the vitality of the testis before testicular torsion. However, from the literature reviewed, we discovered an inconsistency in the results of different studies. After analyzing the case data of 167 children with testicular torsion in our hospital, we observed that, in addition to the duration of symptoms and degree of testicular torsion, monocyte count was also an independent risk factor for testicular salvage [OR = 0.020, 95% confidence interval (CI): 0.001–0.404, *P* = 0.011].

Peretti et al. (17) compared the hematological parameters of 19 patients with testicular torsion (8 cases of orchiopexy and 11 cases of orchiectomy). They noted that MPV was predictive of testicular viability in patients with symptoms lasting <6 h. He et al. (5) found that symptom duration, degree of spermatic cord torsion, and MPV could be used as predictors of testicular viability during testicular torsion. They believe that microthrombosis in the testicular parenchyma after testicular torsion leads to increased platelet activity and massive proliferation. This phenomenon is more significant in the window stage of testicular torsion. However, in our study, MPV was not a risk factor for testicular salvage (*P* = 0.699).

Neutrophils and lymphocytes play vital roles in the occurrence and development of systemic inflammatory responses (SIRs); therefore, NLR can be used to evaluate the progress of SIR-related diseases (18). After analyzing the data of 60 patients with testicular torsion (22 cases of orchiopexy and 38 cases of orchiectomy), Jang et al. discovered that NLR could be used as a predictor of testicular vitality in patients with testicular torsion within 3–12 h (9). However, in this study, although univariate analysis showed that NLR was associated with testicular rescue (*P* = 0.042), multivariate analysis showed that NLR could not be used as an independent risk factor for testicular salvage (*P* = 0.509). Unfortunately, there is no data on monocytes in Jang et al.'s study compared to ours.

Through multivariate logistic regression analysis, Yilmaz et al. observed that monocyte count was the only significant variable of testicular viability in patients with testicular torsion (OR = 0.046, 95%CI: 0.006–0.366, *P* < 0.004) and that monocyte count could predict testicular salvage in patients with testicular torsion (19). Merder et al. (7) retrospectively analyzed the data of 88 patients with testicular torsion (61 cases of orchiopexy and 27 cases of orchiectomy). They observed that symptom duration and monocyte count were predictors of testicular viability. In this study, the monocyte count in the failed salvage group was significantly higher than that in the successful salvage and control groups; the multivariate logistic analysis showed that the monocyte count was an independent risk factor for testicular viability in patients with testicular torsion.

The primary pathophysiological mechanism of testicular torsion is testicular ischemia/reperfusion injury (20). Some animal experiments have shown that oxidative stress and germ cell-specific apoptosis after testicular torsion increase the neutrophils in the testis, promoting the proliferation of immune cells, such as monocytes and lymphocytes, and further aggravating the apoptosis of germ cells (21). Monocytes are important immune cells that can phagocytize and present antigens, secrete chemokines, and proliferate during infection and injury (22). After tissue injury, monocytes proliferate rapidly in the bone marrow and enter the circulation under the action of monocyte chemokine CCL2, which can reach the site of tissue injury within 1 h (23). Monocytes can also differentiate into dendritic cells or macrophages involved in the inflammatory response caused by testicular injury (22, 24). Nemeth et al. (25) examined the hematological parameters of rats after testicular ischemia-reperfusion, and observed that the white blood cell count and monocyte-granulocyte ratio increased significantly, and the monocyte-granulocyte ratio doubled on the first postoperative day. However, research on the mechanism of monocytes after testicular torsion is limited, and more studies are needed to verify the relationship between monocytes and testicular viability.

This study is the most extensive report on the analysis of hematological parameters of testicular torsion in children and confirms the value of monocyte count in predicting testicular viability preoperatively. However, this study had some limitations. First, we defined a 50% reduction in testicular volume compared with the contralateral side as testicular atrophy and considered testicular salvage failure without further consideration of testicular blood flow. Second, the age range of the study participants was wide (1 month to 16 years), which may have led to errors in the results. Finally, we may need to ensure an extended follow-up period in a future study to understand testicular development after orchiopexy.

In conclusion, this study demonstrated that symptom duration, degree of spermatic cord torsion, and monocyte count were predictors of successful testicular salvage. Clinicians can predict testicular survival in patients with testicular torsion before surgery based on the monocyte count. However, there are differences in the reports of predictors of testicular vitality in different studies. Therefore, a prospective study with a larger sample size is required to evaluate the predictive value of monocytes in testicular salvage.

## Data availability statement

The raw data supporting the conclusions of this article will be made available by the authors, without undue reservation.

## Ethics statement

The studies involving human participants were reviewed and approved by Ethics Board of Shenzhen Children's Hospital. Written informed consent to participate in this study was provided by the participants' legal guardian/next of kin.

## Author contributions

PC and SL: conceptualization and visualization. PC and LL: data curation. WH and PC: formal analysis. MS and SL: funding acquisition and methodology. LL and NC: investigation. WH and NC: supervision. GZ and WH: validation. PC: writing—original draft. All authors: writing—review and editing. All authors contributed to the article and approved the submitted version.

## Funding

This study was supported by grants from Shenzhen Fund for Guangdong Provincial High-level Clinical Key specialties (No. SZXK035) and National Natural Science Foundation of China (Grant No. U1904208).

## Conflict of interest

The authors declare that the research was conducted in the absence of any commercial or financial relationships that could be construed as a potential conflict of interest.

## Publisher's note

All claims expressed in this article are solely those of the authors and do not necessarily represent those of their affiliated organizations, or those of the publisher, the editors and the reviewers. Any product that may be evaluated in this article, or claim that may be made by its manufacturer, is not guaranteed or endorsed by the publisher.
